# Emerging Water Quality Problems in Developing Countries

**DOI:** 10.1155/2014/215848

**Published:** 2014-05-12

**Authors:** Manish Kumar, Gurmeet Singh, Tushara Chaminda, Pham Van Quan, Keisuke Kuroda

**Affiliations:** ^1^Department of Environmental Science, Tezpur University, Tezpur, Assam 784028, India; ^2^Jawaharlal Nehru University, New Delhi, India; ^3^Department of Civil and Environmental Engineering, Faculty of Engineering, University of Ruhuna, Galle, Sri Lanka; ^4^Hanoi Architectural University, Vietnam; ^5^Center for Environmental Risk Research, National Institute for Environmental Studies (NIES), 16-2 Onogawa, Tsukuba, Ibaraki 305-8506, Japan

It has been an interesting endeavor for us to contribute as the guest editors for this special issue. At the same time, it was a daunting task for us editors to go through each of the papers thoroughly and select only the ones which we thought were unique and matched the scope of this special edition of the journal.

According to Nobel Laureate Richard Smalley (1996 Noble in Chemistry), water is the second, next to energy, among the humanity's top ten problems in the next 50 years. Water, one of the most important factors required for life to sustain on planet Earth, requires proper attention in terms of uses as well as treatments. The challenge for scientists and engineers working in the field of water is that they encounter new problems with every passing day, where the effective use of new techniques, such as tracers and modelling simulation, is becoming inevitable. The purpose of this special issue is to define the emerging water problems and possible technical solutions to the arising problems. We were interested in articles that explore various water research domains with relevant techniques and integrate in a single text the subject matter that deals with the concurrent “Emerging Water Quality Issues.” We, in principle, wanted to emphasize on topics like (i) emerging water problems in developing countries, (ii) emerging pollutants in water environment, (iii) health-related issues of water environment, (iv) water analysis tools and techniques, (v) flood-related issues, (vi) public involvement and participation, and so forth.

This special issue is comprised of topics dealing with the subject mentioned above and intended for the students, professionals, and researchers working on various aspects of water quality issues. The issue seeks its impact from its diverse topic coverage revealing situations of different contemporary issues, such as drinking water treatment, microbial contamination in fresh water system as well as in sewage water, metal contamination in sediment using isotope tracers, using low cost adsorbent like rice husk, and projections of climate change in Tibetan Plateau. Various case studies have been discussed to present a cutting edge scenario of the problem, perspective, and challenges of emerging water quality issues in the present context of climate change and significant human interventions.

To mention a few highlights of the findings reported in the issues are as follows: H. Sakai et al. have conducted a survey of the quality of the source water and drinking water in urban areas of Myanmar; for this study two urban areas were selected and the drinking water samples were collected from a variety of sources like public pots, nonpiped taps, piped taps, and bottled waters. The overall water quality of the samples was found to be good while As and F^−^ were present at relatively higher levels and should be remediated. The microbial levels were found to be least in the bottled water and highest in pots. This study could be used to devise better solutions for treatment and supply of drinking water in the urban areas of Myanmar.

H. Sakai et al. also conducted a study on the occurrence and distribution of microcystins in Lake Taihu, China. Microcystin is an emerging pollutant and very toxic in nature; it is reported to be carcinogenic in animals including humans. Microcystin levels correlated with COD, chlorophyll-a, and turbidity. This study is important because, unlike traditional pollutants like As or F^−^ in the water, research in the field of microcystin is limited and its effects were not properly understood. Moreover, the study has been carried out in the third largest lake of China, Lake Taihu, which is a unique opportunity to study the effects of this pollutant in a lake ecosystem.

Z. Hao et al. have checked the effectiveness of 22 different global climate models (GCMs) provided by the IPCC to predict the climate change pattern in the Tibetan Plateau. Tibetan Plateau is a very large area and almost all the models which were reviewed for their climate predicting abilities had limitations in predicting the precipitation (which was overestimated) and temperature (which was underestimated). It has been suggested that both the temperature as well as the precipitation levels in the Tibetan Plateau will increase in the next 90 years. Barring a few models, resolution of most of the climate predicting models were low and further work needs to be done in order to improve the accuracy of these models. Based on the studied models, the authors have predicted three possible scenarios of future climate in the Tibetan Plateau, A1B, A2, and B1. In A2 scenario, the greatest increase in precipitation was observed while in the least increase in precipitation was observed in the A1B scenario. This study is important, because it tests the effectiveness of different established GCMs to predict the changes in the climate of a large geographical area. The inputs from this study can be used for further improvement and development of the climate predicting models.

Vedat et al. tested the effectiveness of a hybrid membrane system in removing natural organic matter (NOM); ultrafiltration membranes fortified with powdered activated carbon (PAC) were found to remove NOM more effectively than unfortified UF and microfiltration (MF) membranes. This technique also required less amount of energy as the pressure required for separation of NOM in PAC-UF was lesser than that required in MF and UF membranes. However, the problem with using PAC activated filters was the clogging of the filter pores by the deposited PAC, called membrane fouling.

S. Rungrodnimitchai reported a cost effective method of heavy metal remediation using raw materials widely available in their geographical locality using bagasse and rice husk to effectively sequester Pb^2+^, Cd^2+^, and Cr^2+^ ions in sorption experiments. The bagasse and rice husk were phosphorylated after pretreatment with NaOH to increase their sorption capacity; it was found that these two waste materials could remove the heavy metals more effectively than commercially available ion exchange resins like Dowax. Modified rice husk took lesser time (about 20 minutes) to reach 92% removal of Pb^2+^, while Dowax took 90 minutes to reach the same efficiency. This study is important because it highlights the effectiveness of easily available crop waste like bagasse and rice husk in removal of heavy metals.

A study to determine the effects of nutrient inputs on strophic state and environmental quality of coastal ecosystem has been also included. Both TRIX (Trophic Index) and the AZCI (Arid Zone Coastal Water Quality Index) systems were used to assess the seasonal change in the trophic levels of the Gulf of California. In summer, an oligotrophic condition prevailed due to absence of sewage disposal into the Gulf of Mexico, while in winter there was an increase in the nutrient load of the water in the Gulf, which leads to a mesotrophic condition. Temperature variation between summer and winter in the region is high and water temperature itself varies by ~12°C between summer and winter. This difference would play an important role in the growth of phytoplanktons in the Gulf of California which also affects the overall quality and DO of the water.

It is evident but worth mentioning that all the papers have been prepared by individuals who are experts in their field. Honest effort has been made to check the scientific validity, depth of study, and justification of each chapter through several iterations. We, the editor, publisher, and hard-working water professionals, have put together a comprehensive reference work with a belief that this issue will be of immense use for present and future colleagues who teach, study, research, and/or practice in this particular field.

*Manish Kumar* *Manish Kumar* *Gurmeet Singh* *Gurmeet Singh* *Tushara Chaminda* *Tushara Chaminda* *Pham Van Quan* *Pham Van Quan* *Keisuke Kuroda* *Keisuke Kuroda*

